# Vascular Endothelial Growth Factor Biology and Its Potential as a Therapeutic Target in Rheumatic Diseases

**DOI:** 10.3390/ijms22105387

**Published:** 2021-05-20

**Authors:** Thi Hong Van Le, Sang-Mo Kwon

**Affiliations:** Laboratory for Vascular Medicine and Stem Cell Biology, Convergence Stem Cell Research Center, Medical Research Institute, Department of Physiology, School of Medicine, Pusan National University, Yangsan 50612, Korea; lethihongvan25121978@gmail.com

**Keywords:** VEGF, rheumatic diseases, therapeutic target

## Abstract

Rheumatic diseases constitute a diversified group of diseases distinguished by arthritis and often involve other organs. The affected individual has low quality of life, productivity even life-threatening in some severe conditions. Moreover, they impose significant economic and social burdens. In recent years, the patient outcome has been improved significantly due to clearer comprehension of the pathology of rheumatic diseases and the effectiveness of “treat to target” therapies. However, the high cost and the adverse effects are the concerns and full remissions are not often observed. One of the main processes that contributes to the pathogenesis of rheumatic diseases is angiogenesis. Vascular endothelial growth factor (VEGF), a central mediator that regulates angiogenesis, has different isoforms and functions in various physiological processes. Increasing evidence suggests an association between the VEGF system and rheumatic diseases. Anti-VEGF and VEGF receptor (VEGFR) therapies have been used to treat several cancers and eye diseases. This review summarizes the current understanding of VEGF biology and its role in the context of rheumatic diseases, the contribution of VEGF bioavailability in the pathogenesis of rheumatic diseases, and the potential implications of therapeutic approaches targeting VEGF for these diseases.

## 1. Introduction

The most common rheumatic diseases are characterized by joint inflammation and may involve peri-articular tissues, including tendons, ligaments, bones, and muscles. Other organs, such as the skin, heart, vasculature, eyes, lungs, brain, and intestinal tract may also be affected [1,2]. There are more than 200 degenerative, inflammatory, and autoimmune conditions [3]. Musculoskeletal diseases are one of the top reasons of disability worldwide [4]. The quality of life of affected individuals is lowered significantly. The etiology of rheumatic diseases is often unclear. Additionally, there are many things that need to be clarified in the pathogenesis of rheumatic diseases. So, the therapy treatment in rheumatic diseases has many challenges. Although, in recent decades, the new biologic agents have dramatically changed the prognosis and outcome of some autoimmune arthritis diseases [3]. A clearer understanding of the role of VEGF in skeletal development and growth, also its role of the pathogenesis of rheumatic diseases, may contribute to the development of potentially effective therapies. In this review, we focus on rheumatoid arthritis (RA), osteoarthritis (OA), ankylosing spondyloarthritis (AS), systemic lupus erythematosus (SLE), systemic sclerosis (SSc), and Sjögren syndrome (SS).

Angiogenesis, the development of new vessels from the current ones, is the principal driver of synovitis, the prominent hallmark of rheumatic diseases [5,6]. Nutrients and oxygen supplied via the new vessels augment the inflammatory mass. New vessels forming include several steps. This process occurs when endothelial cells are activated by angiogenic factors that are released during angiogenesis. Subsequently, a series of events happen including proteolytic enzyme secretion, extracellular matrix degradation, endothelial cell migration, and new basement formation, respectively [7].

VEGF participates in almost all steps of angiogenesis [8]. Emerging evidence has shown that VEGF contributes significantly to the pathogenesis of many disorders such as RA, autoimmune diseases because of its role in angiogenesis [5]. Several therapies targeting the VEGF pathway have been used in cancers and diabetic retinopathy treatment [9]. In several rheumatic diseases, anti-VEGF therapies or in the combination with current treatment are experimented in the animal disease model and the results of these studies are promising. We reviewed our present knowledge of VEGF biology and its relationship with the pathogenesis of rheumatic diseases by collecting some data sources, including basic studies, clinical studies, and clinical trials with a particular focus on RA, AS, OA, SLE, SSc, and SS.

## 2. VEGF Biology and Its Role in Musculoskeletal Physiology

### 2.1. VEGF Biology

In 1983, Senger et al. first isolated and defined VPF (vascular permeability factor), an original name of VEGF. VEGF is a homodimeric protein with 34–42 kDa in molecular weight [10]. From this, many studies contribute to a clearer understanding of the structure, physiological activities, and its role in pathological conditions. Vascular endothelial growth factor (VEGF, also known as VPF) function as a mitogen specifies in endothelial cells [11]. The role of VEGF in physiological and pathological activities is no doubt. VEGF activity is not limited to the vascular system; VEGF also participates in other physiological activities related to the growth of the fetus, bone, and reproductive system [12,13]. Moreover, VEGF plays an important role in the development of various diseases including tumors forming, hematologic cancer, ocular diseases in diabetes, inflammation, brain edema, and a group of obstetrics and gynecology diseases such as polycystic ovary syndrome, endometriosis, and preeclampsia [12].

In mammals, VEGF-A, -B, -C, -D, and placental growth factor (PlGF) are the members of the VEGF family. VEGF-A has been described as a central regulator of angiogenesis and currently its data are the largest compared to other VEGF members [12]. VEGF121, VEGF165 (VEGF164 in mice), VEGF189, and VEGF206 are its various isoforms, and the major characteristic that differentiates the isoforms is their affinity for heparin [13,14,15]. VEGF-B strongly expresses in the heart and muscle and contributes to the proliferation of endothelial cells [16], while lymphangiogenesis is regulated by VEGF-C and -D [17].

VEGF receptors consist of distinguished tyrosine kinases (RTKs), VEGFR-1 (Flt-1), VEGFR-2 (KDR/Flk-1), and VEGFR-3 (Flt-4). Their expression varies in various tissues, a pathological condition. There is a difference in the binding position of every isoform. VEGF-A binds to VEGFR-1 and VEGFR-2. VEGF-B and PlGF bind to VEGFR-1. VEGF-C and VEGF-D bind to VEGFR-2 and VEGFR-3 [12]. VEGF-A was initially described as a VPF [10] and shows the role in angiogenesis and mitogenesis [18]. Neuropilin-1 (NRP1) and -2 (NRP2) are co-receptors of VEGFR-1. NRP1 serves as a receptor and binds to the collapsin-semaphorin family, and is required for vascular development [19]. Soker et al. showed that NRP1 is found on endothelial cells and tumor cell surfaces and binds to VEGF165. NRP-1 null mice showed embryonic lethality, suggesting that NRP1 is an essential factor in the growth of the vascular system [20].

VEGF is produced by many types of cells, including fibroblasts [21], macrophages [22], endothelial cells [23], neutrophils [24] and T cells [25]. Various factors regulate VEGF expression including oxygen tension, growth factors, oncogenes, cytokine and cell-bound stimuli, and in turn by VEGF-driven signaling [26,27,28]. Low oxygen tension induces VEGF mRNA expression in various pathophysiological conditions. Subsequently, HIF-1, a central regulator of hypoxia response, regulates the VEGF expression [29]. Several growth factors provoke VEGF mRNA expression including epidermal growth factor (EGF), transforming growth factor-α, transforming growth factor-β (TGF-β), keratinocyte growth factor, insulin-like growth factor, and cytokines such as interleukin (IL)-1 and IL-6 also contribute to the VEGF regulation [13,26].

The understanding of VEGF has seen significant achievement in recent decades, contributing to the development of new therapies in some special diseases such as cancer or retinopathy. To date, many drugs targeting VEGF pathways have been indicated in a variety of disease conditions with desired effects. In 2004, the first agent approved by the FDA was bevacizumab, a humanized form of anti-VEGF Ab, indicated in metastatic colon cancer [30], and 2 years later, ranibizumab, a recombinant antibody fragment from human, showed the effectiveness in intraocular for age-related macular degeneration treatment [31], Ziv-aflibercept (targets: VEGF-A, VEGF-B, and PlGF), ramucirumab (target: VEGFR-2), and multiple tyrosine kinase inhibitors are indicated in combination with other therapies for various cancers [32]. Pegaptanib, an RNA aptamer, directs against VEGF with a high affinity with the target molecule [33]. In a recent study, faricimab, an antibody that inhibits both VEGF-A and Ang-2, was also shown to be effective and safe in diabetic macular edema in phase II clinical trials [34]. The barrier of anti-VEGF therapies is the concerns about the side effect. The adverse effect of this therapy also reduces considerably by local administration because of the dramatically lower dose compared to systemic administration. Thus, this is a crucial suggestion for various disease studies such as arthritis [30,35]. Currently, several ongoing studies and human clinical trials have focused on VEGF pathways and their combination with other therapies in numerous diseases.

### 2.2. VEGF in the Musculoskeletal System

Rheumatic diseases are inflammatory diseases that involve various organs and present articular and extra-articular manifestations. Many studies have shown the role of VEGF in various activities of the musculoskeletal system in physiology, pathology condition, and other processes related to the pathogenesis of rheumatic diseases. Besides the uncontroversial role of VEGF in angiogenesis during fetus, organ development, VEGF also showed its crucial role in other aspects of synoviocytes, bone development including endochondral ossification, osteoblast, and osteoclast differentiation. Synovium is a part of the structure of a joint that is a remarkable injury position in rheumatic diseases. VEGF-NRP1 axis regulates the synoviocyte apoptotic process [36]. One of the fundamental processes in the bone development and growth is the process that cartilage is replaced by bone, called endochondral ossification. VEGF also showed the material regulator of this process [37]. In mice, chondrocytes survival and differentiation require the role of VEGF [38]. VEGF expression increases during osteoblast differentiation. VEGF enhances the proliferation of osteoblast [39,40]. Increased bone resorption, one of the characteristics of almost all rheumatic diseases, causes permanent injuries in the bone. VEGF may increase bone resorption by increasing osteoclast activity through its action on VEGFR-1 [41], VEGFR-2 signaling [42,43]. This may be mediated via the interaction of VEGFR and receptor activator of nuclear factor kappa-B ligand [41]. Nakagawa et al. showed that mature osteoclast survival requires the VEGF [43]. Immune cells play an important role in the pathology of autoimmune arthritis, and dysregulation of the immune response is a prominent feature. VEGF directly affects immune cells [44]. VEGF affects inflammatory processes in various ways. In lymphatic endothelial cells, this study observed VEGFR-3 expression and it generates the main signal for lymphangiogenesis. VEGFR1 also exist in the macrophages that produce the cytokine/chemokine. This VEGFR-1-macrophage axis stimulates non-inflammatory and inflammatory responses in arthritis [45]. In RA and OA, synovium showed that HIF-1 and HIF-2, the essential regulators of VEGF signaling, express abundantly [46]. Taken together, VEGF appears to be a considerable factor that contributes to the pathogenesis and various aspects of rheumatic diseases.

## 3. VEGF in RA

RA is a systemic inflammatory disease characterized by not only chronic synovial membrane (SM) inflammation, cartilage damage, bone erosion, but also other organ damages such as the skin, heart, lungs, and eyes. The incidence of this disease in the general population is about 1% with the highest proportion in middle age. Women have a higher incidence of this disease. The pathogenesis of RA is complicated with the role of many cells including immune cells, fibroblast, chondrocyte, dendritic cells. RA can lead to disability, inferior quality of life, and increased comorbidities. The disease progression and prognosis have been improved considerably by new biologic agents including TNFα inhibitors, IL6 inhibitors, T-cell blockers, Janus kinase inhibitors, in combination with standard therapy [47,48,49].

The SM is rich in blood vessels [50], and numerous factors have been implicated in increased SM vascularization, including VEGF, TGF-β, and FGF [51]. There is an increase in blood vessel number of synovium and endothelial proliferation in RA. In an early stage of RA, endothelial activation and synovium hyperplasia are detected. Inflammatory cells infiltrate increasingly to SM through new blood vessels during the progression of RA [52]. Taken together, in RA, promoting and maintaining synovial hyperplasia requires angiogenesis. VEGF, a proangiogenic factor, plays an essential role in synovial angiogenesis [53], a key process during the development and progression of RA. Synovial angiogenesis reduces in VEGF knockout mice using antigen-induced models of arthritis [54], which suggests that VEGF contributes to the pathology of RA. VEGF and its receptors are detectable in serum and synovial fluids. VEGF expression is observed in synovial macrophages and fibroblasts of RA patients [55,56,57,58,59,60]. Moreover, several studies have reported that SM endothelial cells and cells of the lining layer also high express in RA. VEGF regulates the migration and proliferation of endothelial cells in RA [58,59]. In RA synovial tissue, VEGF-A and their receptors express higher compared to normal tissue [60]. Kim et al. showed that VEGF-NRP also prevents synoviocyte apoptosis by inducing Bcl-2 expression and translocation of Bax [36]. This leads to synovial hyperplasia called “pannus”, which is a prominent characteristic of RA. Bone erosion is another crucial characteristic of RA disease. Bone destruction depends on the osteoclast differentiation and VEGF also has a role in this process in RA. VEGF impact on osteoclast differentiation both directly regulates osteoclast differentiation from monocytes and induces fibroblast-secreted RANKL (nuclear factor-kappa-B ligand), a central cytokine in osteoclast differentiation [61]. Additionally, VEGF is also associated with disease activity as well as other markers. VEGF concentration was found to be significantly different in various rheumatic diseases, including RA, SLE, antiphospholipid syndrome, and mixed connective tissue disease. VEGF was expressed at the highest concentration among these diseases in that study [62]. VEGF concentration in the serum increases and correlates with disease activity, C-reactive protein level, and radiographic progression [63,64]. In clinical studies, VEGF was used as a marker to assess treatment response. Synovial VEGF expression, as well as synovial vascularization, decrease significantly in RA treatment with infliximab in the combination therapy with methotrexate [65]. High VEGF levels strongly correlate with RA pathogenesis. Another angiogenic cytokine, PlGF, represents the synovitis severity of RA as assessed by ultrasound [66]. Recently, some studies reported that serum VEGF is more valuable than traditional factors such as CRP in determining the treatment response of patients receiving biologic DMARDs [66,67]. In the management of RA, the optimal goal is prevention of permanent injuries in a joint, so the diagnosis in the early stage of the disease is key. Assessment by biomarker may be more sensitive compared to disease activity score in this stage [67]. Additionally, VEGF-C gene polymorphisms may contribute to susceptibility, potential diagnostic markers, and therapeutic targets in patients with RA [68]. Studies have shown that several pathways are related to VEGF in RA. Expression and secretion of VEGF in RA may occur via the IL-6/JAK2/STAT3/VEGF pathway [69]. VEGF/Ang2-induced proangiogenic/inflammatory mechanisms are mediated by Notch signaling pathways in RA [70]. This suggests that the VEGF/Ang2-Notch and IL-6/JAK2/STAT3/VEGF axes may be potential therapeutic targets.

The effect of anti-VEGF therapy or its combination with current therapy was investigated in animal models. In a murine model of collagen-induced arthritis, Miotla et al. used soluble VEGF receptor for collagen-induced arthritis treatment and showed that the disease severity decreased in this treatment group [71]. Other studies also showed the consistency in the effectiveness of anti-VEGF polyclonal antibodies in collagen-induced arthritis model [72,73]. A study used VEGF blockade monotherapy in the comparison with current monotherapies such as tocilizumab or methotrexate with promising results. This study compared the effect of ranibizumab (anti-VEGF antibody) and tocilizumab (interleukin-6R antagonist) in rat adjuvant-induced arthritis by intra-plantar injection. The effects on inflammatory, angiogenesis, apoptosis inhibition were observed in both groups. Interestingly, anti-VEGF reduces the bone and cartilage destruction compared to methotrexate or tocilizumab treatment [74]. Another anti-VEGF signaling agent, Ramucirumab, is a monoclonal antibody against the VEGFR2, and co-therapy with methotrexate showed synergistic effects in a RA experimental model. Ramucirumab is also used by intra-plantar injection [75]. Recent evidence suggests that anti-VEGF therapy by intra-articular injection may be an option for monotherapy or the combination with standard therapy. Further studies are required to evaluate the effect or anti-VEGF therapy in RA treatment.

## 4. VEGF in AS

Seronegative spondyloarthritis group consists of AS, reactive arthritis, psoriatic arthritis, enteropathic arthropathy, undifferentiated spondyloarthropathy. AS is the most common of the seronegative spondyloarthritis diseases, with onset at a young age; it may cause disability if undiagnosed and untreated. AS is a chronic progressive disease. Clinical symptoms include low back pain, sacroiliitis, enthesitis, spinal ankylosis, and deformity. Inflammatory bowel disease, acute anterior uveitis, psoriasis, cardiovascular disease, iritis, and pulmonary involvement are other manifestations [76]. Current treatments for spondyloarthritis are anti-inflammation drugs and several biological agents. Targeted therapy includes TNF-α inhibitors (etanercept, infliximab, golimumab, adalimumab, and certolizumab pegol), IL-12/23 inhibitors (ustekinumab), and IL-17 inhibitors (secukinumab and ixekizumab) [77]. Treatment with anti-TNF-α inhibitors has been proven to be an important strategy; however, its effect on the radiographic progression of the disease remains unclear. Therefore, improved understanding of AS pathology will contribute to targeted and combination therapies for AS treatment.

Diagnosis of early inflammatory arthritis significantly improves patient outcomes, because the permanent injuries can be prevented. Besides joint injuries such as bone erosion in synovial joints, AS patients suffer new bone formation and new cartilage was followed by calcification [78,79] that lead to syndesmophytes, anthesophytes, spine ankylosis, these damages cannot recover. The mechanism of this process remains unclear. Angiogenesis is one of the hypotheses to explain about the new bone formation in AS. Not only new bone formation but also sacroiliitis, and enthesitis that are various manifestations of AS, require angiogenesis. VEGF is a central regulator of this process [80]. Fearon et al. observed that VEGF is also expressed in early inflammatory arthritis and is closely related to angiopoietins [81]. It suggests that VEGF may be a potential factor that regulates these events in the pathogenesis of early AS. VEGF may directly regulate osteoblast differentiation from synovial fibroblast that contributes to the new bone formation in AS [82]. Moreover, VEGF may stimulate the COX2 pathway [83]. VEGF also participates in inflammatory in AS [84]. Thus, VEGF may be a target for the treatment of arthritic diseases. Several studies showed that macrophages in SM and entheses of AS secrete VEGF [82,85,86]. Thus, this suggests that VEGF has various functions apart from angiogenesis. VEGF is also a factor to assess the progression, prediction, and susceptibility of this disease in numerous studies. There is a significant increase in serum VEGF levels in AS patients compared to healthy persons [87,88,89]. VEGF concentration also has a difference in the serum and synovial fluid. In AS patients with peripheral arthritis, VEGF levels are lower considerably in synovial fluid compared to serum levels [88]. Additionally, some evidence showed that VEGF correlates with clinical manifestations such as peripheral arthritis [88] and Bath Ankylosing Spondylitis Disease Activity Index by evaluation the fatigue, spinal pain, joint pain, enthesitis, morning stiffness [84,88,90], other markers of inflammatory [84,91], and duration [89]. However, extra-articular manifestations, syndesmophytes, or the severity of sacroiliitis do not register the association with VEGF [84,89]. Therefore, serum VEGF may be considered a marker of disease activity in AS [84,88,92]. However, another study supported that VEGF only shows a part of the disease progression [93]. Various isoforms of VEGF have also shown inconsistent results. For example, in a study by Bandinelli et al., the levels of VEGF-C did not show a correlation with disease activity [94]. Prediction through spinal radiographic progression serves as the main factor in the prognosis and treatment choice of AS, although it is challenging. VEGF may better predict bone damages in patients with axial spondyloarthritis [88,93,95]. In contrast, Braun et al. did not show any correlation between VEGF and disease activity score based on clinical manifestations and abnormal signs in magnetic resonance imaging. [96]. However, VEGF in combination with other factors such as biomarkers, clinical characteristics, predict better for radiographic progression in axial spondyloarthritis [97]. TNFα inhibitors are the biologic agents that are indicated frequently in AS. VEGF levels also significantly decrease following anti-TNFα therapy [91,98,99]. VEGF also correlates with bone mineral density in AS treatment with infliximab [99]. VEGF levels decrease sharply in AS treated with secukinumab (Anti-IL-17A monoclonal antibody), and decreases in VEGF levels also correlate with inflammatory osteogenic biomarkers [100]. Additionally, VEGF also associates with extra joints and spine manifestations in spondyloarthritis such as subclinical gut inflammation. VEGF and PIGF levels markedly increased in the intestinal mucosa of AS patients without any history of gut inflammation compared to healthy controls [101]. Several studies also support that VEGF is related to AS susceptibility [87], suggesting that VEGF plays a role in the pathogenesis of AS.

A study assessed the effect of anti-VEGF therapy in AS mouse model of proteoglycan-induced arthritis by injection of soluble fms-like tyrosine kinase-1 (sFlt-1), a secretory decoy receptor for VEGF. It supported that disease activity improves significantly in the group treatment compared to the control group [102]. Currently, the main therapies for AS include non-steroid drug and anti-TNFα, both of them target in inflammatory process with limitations remaining in radiograph progression prevention. Lacout et al. suggested that bone homeostasis should be targeted in AS treatment. Anti-VEGF such as bevacizumab may be a potential therapy for severe disease activity and fast progression [103].

## 5. VEGF in SLE

SLE is the most typical autoimmune disease with various autoantibodies detecting. Other organ damages include skin, hair, blood, nervous system, kidney, vascular, lung, and musculoskeletal manifestations. Women of childbearing age are a high incidence [104]. The etiology of SLE is unclear; many factors are mentioned comprising the gene, ethnic, immune regulatory, hormone, and environment. SLE has many severe complications that lead to life-threatening of patients. In recent decades, the outcome of SLE patients improved considerably because of new immunosuppressant agents [105].

VEGF levels are associated with SLE risk, active SLE risk, lupus nephritis risk [106], and the risk of SLE in a Chinese Han population related to VEGFR1 gene polymorphisms [107]. VEGF is an independent predictor of disease activity [106,108]. In pediatric-onset SLE, VEGF was shown to be one of the six markers of endothelial dysregulation. Therefore, VEGF may be a potent biomarker for pediatric-onset SLE activity as well as organ involvement. It is also helpful for understanding vascular pathogenesis and disease monitoring [109]. VEGF associates with the incidence of clinical manifestations of SLE. Oral ulceration is a common symptom in SLE patients that has a relationship with VEGF [109]. Additionally, VEGF may be contributed to the raised frequency of neuropsychiatric disorders in SLE patients [110]. Female SLE patients in pregnancy often progress more severely with active SLE and various complications to both mother and fetus. Preeclampsia also is an obstetrics disease in pregnancy. To answer the SLE patients in the acute phase of SLE with nephritis or inactive SLE or preeclampsia is challenging to clinicians due to overlapping symptoms. Recent publications showed that there is a significant difference in VEGF level between preeclampsia, inactive SLE, and active lupus nephritis. So, this result suggests that VEGF level may be a useful marker to clinicians [111]. Lupus nephritis is a severe manifestation and prognosis in patients with SLE. VEGF-A participates in the pathogenesis of proliferation lupus nephritis via its impact on the relationship of endothelial cells and epithelial cells [112]. VEGF levels relate to the development of kidney damage [106]. Another study also showed that there is a considerable increase of VEGF in lupus nephritis compared to the non-lupus nephritis and the control group. However, there was no statistically significant relationship between serum VEGF levels and the histological classes of lupus nephritis [113]. Thus, VEGF may be contributing to the pathogenesis of these manifestations. Furthermore, there was a decrease in the level of VEGF-A in SLE patients taking mycophenolate mofetil compared to the non-taking mycophenolate mofetil group [114]. Another study observed that the level of VEGF-R2 significantly decreased in a group of SLE patients treated for a long time compared to the group of newly diagnosed, untreated SLE patients. Various drugs, including prednisone, immunosuppressive drugs were used in this study [115]. VEGF correlates with disease activity [116,117,118].

## 6. VEGF in OA

OA is the most common joint disorder in middle age and elderly people. OA affects all parts of joints, including cartilage, subchondral bone, synovium, ligaments, and periarticular muscle. Cartilage degeneration is fundamental damage [119]. OA is the principal cause of disability and pain [120]. Disease-modifying drugs are currently not available, and therapy is mainly aimed at symptom relief [121].

Angiogenesis forms a network of neovascularization in synovium called OA pannus. Angiogenesis associates with osteophytes formation, VEGF promotes this process [122]. Hyperangiogenesis contributes to synovial inflammation and microstructural deterioration during OA [70]. Augmentation of VEGF signaling exacerbates joint OA formation by increasing osteoclast differentiation, metalloproteinase, and chondrocyte apoptosis leads to bone and cartilage destruction [123]. There is increased expression of VEGF in the entire structure of joints, synovial fluid in OA. Its receptors strongly express in OA chondrocytes. Similarly, VEGF also registers the increase in serum and synovial fluid [124,125,126]. The role of VEGF is observed in various processes of bone homeostasis. VEGF enhances osteoclast differentiation, osteoclast survival, RANKL secretion [123]. There is an increase of VEGF levels in osteoblasts from patients who replaced total hip [127]. VEGF can be used as a marker to monitor the response to OA therapy [128]. Pain is the key symptom of OA and the mechanism of pain in OA is not completely understood; therefore, a clear understanding of the pathogenesis of pain in OA is essential for the development of targeted therapies. Tanako et al. supported that in human knee OA, VEGF experienced a positive correlation with pain score [129] and Galballa et al. also showed that VEGF not only correlates with pain but also in clinical and radiology symptoms [130]. One of the factors that contributes to the mechanism of pain is the growth of sensory nerves. This process is stimulated by proangiogenic factors [131]. Pain hypersensitivity is a phenomenon that is observed in OA. Several studies suggest that VEGF signaling has an association with this condition [132,133,134]. VEGF also increases the expression of several types of MMP, especially MMP-13, key factor results in cartilage degeneration [123,135]. Moreover, hypoxia, a crucial mediator of the VEGF axis, contributes to temporomandibular joint osteoarthritis and accelerates the angiogenesis of condylar cartilage through the HIF-1-VEGF-Notch signaling pathway. HIF-1α and Notch may be novel therapeutic targets for temporomandibular joint osteoarthritis [136]. The accumulated evidence shows that VEGF signaling participates in various aspects of OA including synovitis, mechanism of pain, bone and cartilage destruction.

In vivo studies have also shown positive results with VEGF-targeted therapy. In a rabbit OA model due to trauma, there is a significant decrease in pain, cartilage destruction, synovium inflammatory in group treatment with bevacizumab, a VEGF blocker by intra-articular injection. Moreover, they do not register any adverse effect [137]. Oral administration of a VEGFR-2 kinase inhibitor attenuated OA progression in a mouse model of post-traumatic human knee OA [38]. Anti VEGF signaling seems to be a potential therapy in OA and using anti-VEGF agents by local administration may show more benefits.

## 7. VEGF in SSc

SSc is a rare autoimmune disease marked by the fibrosis of the skin, and involvement of internal organs, especially the vascular system, lung, kidney, and gastrointestinal system and is caused by excessive collagen deposition, immunological disturbances, and accompanying vascular changes. Genetic, environmental, vascular, autoimmunological, and microchimeric factors contribute to the pathogenesis of SSc. The cause of this disease is unknown and the limitation in the treatment therapies leads to the poor outcome of SSc patients. Thus, the tools for early diagnosis and effective intervention are required. A better understanding of the pathology of SSc may contribute to potentially effective therapies to improve SSc patient outcomes. Microvascular damage and dysfunction of angiogenesis are identified abnormalities. Tissue fibrosis results from series events including endothelial dysfunction, inflammatory, increased vascular permeability, platelet aggregation [138,139,140]. Impaired angiogenesis is detected in the digit ulcers of SSc patients [141]. There is a significant increase in serum VEGF levels in both the early and established stages of SSc [142,143]. Jinin et al. suggested that VEGFR-2 level may be an indicator for microangiopathy and helpful for diagnosis in SS [144]. Avouac et al. reported that VEGFR-1 level decreased in SSc patients compared to those in the healthy control. There is a significant increase of VEGF and its receptor expression in skin lesion from SSc patients [144,145].

Pulmonary fibrosis, pulmonary hypertension, and renal involvement are the main causes of mortality. Serum VEGF levels may act as biomarkers for interstitial lung involvement [146]. VEGF does not correlate with intrarenal stiffness and renal function in patients with SSc [147]. VEGF, pigment epithelium-derived factor (PEDF) levels, and the VEGF/PEDF register considerable changes, and they seem to be useful tools for disease activity assessment [148]. However, some studies show that the relationship of VEGF with pulmonary pressure is still unclear [148,149]. VEGF is also used as a marker in combination with other factors to predict disease activity in SSc [143]. VEGF and Ang/Tie2 dysfunction lead to peripheral microvasculopathy in SSc [150]. Nintedanib inhibits simultaneously three types of receptors including the platelet-derived growth factor, fibroblast growth factor, and vascular endothelial growth factor tyrosine kinase, and is approved for idiopathic pulmonary fibrosis. Huang et al. assessed the effect of nintedanib in a mouse model of SSc with promising results. The pulmonary arterial hypertension, microangiopathy destruction, pulmonary, myocardial, and dermal fibrosis are improved in group treatment [151]. Taken together, the inhibitors targeting VEGF signaling may be a potential therapy for SSc treatment.

## 8. VEGF in SS

Primary SS is an autoimmune disease characterized by chronic inflammation that results in dryness of the eye, mouth, and an increased the size of parotid gland. The incidence rate is 0.2–0.5% of the adult population. SS is the most common in middle-aged women [152]. The risk of dental caries and oral infections results in an inferior quality of life. Angiogenesis represents a novel potential marker for primary SS and may contribute significantly to the pathogenesis [153]. VEGF-C expressed in epithelial cells and inflammatory cells of primary SS. There is a considerable increase in VEGFR-3 expression in lymphatic vessels. In primary SS, this study showed that the increased expression of VEGF-C correlates with immune cells, cytokine, and lymphatic EPC originated from bone marrow [154]. Furthermore, the role of VEGF is also supported by another study that showed that tumor necrosis factor alpha converting enzyme (TACE) in TACE/VEGF-R2/NF-κB dysregulation participates in SS pathogenesis [155]. However, the role of VEGF in SS remains controversial, because another study did not show any change in VEGF levels in SS patients. Detection of serum VEGF is less sensitive than detection of VEGF in saliva but the role of saliva VEGF in pathogenesis of SS is unclear [156]. With the evidence limitation, the function of VEGF in SS remains relatively modest.

## 9. Summary

**Figure 1:**
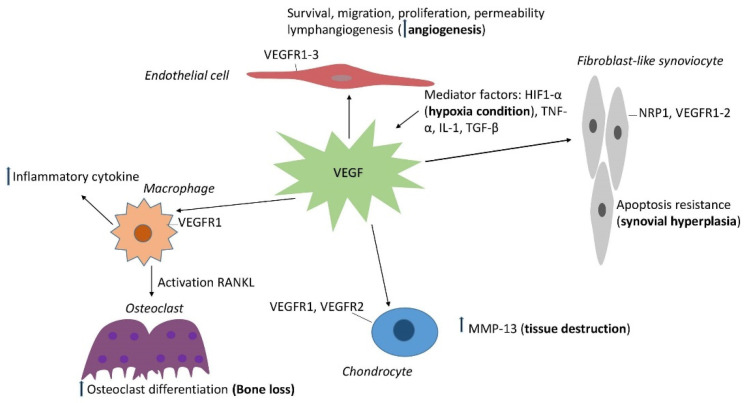
Schematic diagram of the role of VEGF in the development of joint injuries in rheumatic diseases. VEGF plays an important role in angiogenesis, the prominent characteristic of arthritis. VEGF and its receptor strongly express in synovial tissue, fluid. VEGF-NRP1 axis leads to apoptotic resistance of synoviocytes. VEGFR-1 is expressed on the membrane of macrophages. VEGF axis regulates the inflammatory process by cytokine production as well as enhanced bone resorption via increased osteoclast differentiation. In OA, VEGF may increase the expression of MMP-13, leading to tissue destruction.

Taken together, VEGF shows its role in the pathogenesis of many diseases in rheumatology. VEGF is a marker in disease activity assessment and treatment following. The function of VEGF in rheumatic diseases is summarized in Table 1. VEGF contributes to various aspects of the pathogenesis of joint damage in rheumatic diseases including angiogenesis, synovitis, inflammatory, osteoclast differentiation and cartilage degradation. Interestingly, VEGF reveals the association with the mechanism of pain in OA. The role of VEGF in the development of joint injuries is illustrated in Figure 1.

Targeting angiogenesis via inhibition of VEGF may be a potential therapy in rheumatic diseases. However, to minimize the drawbacks of anti-VEGF therapy, appropriate clinical trials are needed to test its safety, efficacy, cost-effectiveness, and methods need to be developed to control the binding ability of drugs to targeted injuries. Based on current evidence, anti-VEGF therapy may be most likely adjunctive therapy for arthritis. Local administration shows more beneficially because of low dose requirement and adverse effect minimizing. In the SSc mouse model, VEGF inhibitor also shows its effect in the improvement of pulmonary hypertension, skin, and lung fibrosis. More studies are needed to clarify the role of VEGF and the effect of anti-VEGF therapy in rheumatic diseases.

## Figures and Tables

**Table 1:** The role of VEGF in rheumatic diseases.

Rheumatic Diseases	Mechanisms	Clinical Results
Rheumatoid arthritis	Regulate the migration, proliferation of endothelial cells Prevent the synoviocyte apoptosis Regulate osteoclast differentiation, induce RANKL secretion IL-6/JAK2/STAT3/VEGF VEGF/Ang2-Notch	High levels Correlate with disease activity, C-reactive protein, radiographic progression VEGF-C contribute to the susceptibility Treatment therapy reduces VEGF expression
Ankylosing spondylitis	Regulate osteoblast differentiation Stimulate COX2 pathway Participate in inflammatory	Correlate with peripheral arthritis, BASDI, inflammatory markers, duration Predict bone damage Correlate with bone mineral density in infliximab treatment Associated with subclinical gut inflammation Anti TNFα, anti-IL-17A decreased serum VEGF levels
Systemic lupus erythematosus	Endothelial dysfunction	High level in lupus nephritis Associated with SLE risk, active SLE, lupus nephritis risk Predictor of disease activity Associated with oral ulceration, neuropsychiatric disorders Correlate with disease activity MMF decreased the VEGF-A levels.
Osteoarthritis	Promote the neovascularization in synovium Increase osteoclast differentiation, osteoclast survival, RANKL secretion Increase metalloproteinase Increase chondrocyte apoptosis Accelerated angiogenesis via HIF-1-VEGF-Notch	Associated with pain, clinical and radiology symptoms
Systemic sclerosis	VEGF/Ang/Tie2 dysfunction	High levels Associated with microangiopathy Biomarker for interstitial lung involvement Combination with other factors to predict disease activity
Sjögren syndrome	TACE/VEGF-R2/NF-κB dysregulation	Remain unclear

## Abbreviations

VEGF	Vascular Endothelial Growth Factor
RA	Rheumatoid arthritis
OA	Osteoarthritis
AS	Alkylosing spondyloarthritis
SLE	Systemic lupus erythematous
SM	Synovial membrane
SSc	Systemic sclerosis
SS	Sjögren syndrome
PIGF	Placental growth factor
MMPs	Matrix metalloproteinases
VPF	Vascular permeability factor
RANKL	Nuclear factor-kappa-B ligand
RTKs	Receptor tyrosine kinases
NRP	Neuropilin
HIF	Hypoxia-inducible factors
TGF-β	Transforming growth factor-β
EPCs	Endothelial precursor cells
PEDF	Pigment epithelium-derived factor
TACE	Tumor necrosis factor alpha converting enzyme
